# Different relationship between ANGPTL3 and HDL components in female non-diabetic subjects and type-2 diabetic patients

**DOI:** 10.1186/s12933-016-0450-1

**Published:** 2016-09-13

**Authors:** Dong Zhao, Long-Yan Yang, Xu-Hong Wang, Sha-Sha Yuan, Cai-Guo Yu, Zong-Wei Wang, Jia-Nan Lang, Ying-Mei Feng

**Affiliations:** 1 Beijing Key Laboratory of Diabetes Prevention and Research, Department of Endocrinology, Lu He Hospital, Capital Medical University, Beijing, 101149 China; 2 Stem Cell Institute, University of Leuven, 3000 Louvain, Belgium

**Keywords:** Angiopoietin-like protein, High-density lipoproteins, Diabetes, Apolipoproteins, Serum amyloid A, Cholesterol efflux

## Abstract

**Background:**

Angiopoietin-like protein 3 (ANGPTL3) is a major lipoprotein regulator and shows positive correlation with high-density lipoprotein-cholesterol (HDL-c) in population studies and ANGPTL3 mutated subjects. However, no study has looked its correlation with HDL components nor with HDL function in patients with type 2 diabetes mellitus (T2DM).

**Methods:**

We studied 298 non-diabetic subjects and 300 T2DM patients who were randomly recruited in the tertiary referral centre. Plasma levels of ANGPTL3 were quantified by ELISA. Plasma samples were fractionated to obtain HDLs. HDL components including apolipoprotein A-I (apoA-I), triglyceride, serum amyloid A (SAA), phospholipid and Sphingosine-1-phosphate were measured. HDLs were isolated from female controls and T2DM patients by ultracentrifugation to assess cholesterol efflux against HDLs. A Pearson unadjusted correlation analysis and a linear regression analysis adjusting for age, body mass index and lipid lowering drugs were performed in male or female non-diabetic participants or diabetic patients, respectively.

**Results:**

We demonstrated that plasma level of ANGPTL3 was lower in female T2DM patients than female controls although no difference of ANGPTL3 levels was detected between male controls and T2DM patients. After adjusting for confounding factors, one SD increase of ANGPTL3 (164.6 ng/ml) associated with increase of 2.57 mg/dL cholesterol and 1.14 μg/mL apoA-I but decrease of 47.07 μg/L of SAA in HDL particles of non-diabetic females (p < 0.05 for cholesterol and SAA; p < 0.0001 for apoA-I). By contrast, 1-SD increase of ANGPTL3 (159.9 ng/ml) associated with increase of 1.69 mg/dl cholesterol and 1.25 μg/mL apoA-I but decrease of 11.70 μg/L of SAA in HDL particles of female diabetic patients (p < 0.05 for cholesterol; p < 0.0001 for apoA-I; p = 0.676 for SAA). Moreover, one SD increase of ANGPTL3 associated with increase of 2.11 % cholesterol efflux against HDLs in non-diabetic females (p = 0.071) but decrease of 1.46 % in female T2DM patients (p = 0.13) after adjusting for confounding factors.

**Conclusions:**

ANGPTL3 is specifically correlated with HDL-c, apoA-I, SAA and HDL function in female non-diabetic participants. The decrease of ANGPTL3 level in female T2DM patients might contribute to its weak association to HDL components and function. ANGPTL3 could be considered as a novel therapeutic target for HDL metabolism for treating diabetes.

**Electronic supplementary material:**

The online version of this article (doi:10.1186/s12933-016-0450-1) contains supplementary material, which is available to authorized users.

## Background

High-density lipoprotein (HDLs) process multiple anti-atherogenic and anti-diabetic properties including reverse cholesterol transport [1], maintenance of endothelium integrity [2, 3], suppression of inflammation [4, 5], promoting pancreatic β cell survival [6] and insulin secretion in response to glucose [7, 8]. In peripheral tissues, infusion of reconstituted HDL (rHDL) containing apolipoprotein A-I (apoA-I), the major component of HDLs, stimulates glucose uptake in both insulin dependent and independent manners [9, 10]. However, type-2 diabetic patients are always accompanied with dyslipidemia, which is featured as high plasma triglyceride concentration [11], decreased high-density lipoprotein-cholesterol (HDL-c) level [12] as well as HDL dysfunction [13].

Accumulated evidence indicates that HDL compositions are key determinants for the atheroprotective functions of HDL. For instance, infusion of reconstitute HDL containing apoA-I restores endothelial cell function in hypercholesterolemia men [14], improves cholesterol efflux [15], inhibits platelet aggression for thrombus formation and reduces neutrophil adhesion to a fibrinogen matrix in diabetic subjects [5]. In contrast to apoA-I, serum amyloid A (SAA) are major acute-phase reactants in inflammation. Upon secretion, SAA are found associated with HDL particles and impair HDL-mediated reverse cholesterol transport [16–19]. Sphingosine-1-phosphate (S1P) is another component in HDLs. Glycation decreases S1P content in HDLs of type-2 diabetic patients whereas adding S1P to diabetic HDLs promotes cardiomyocyte survival when challenged with oxidative stress [20].

Angiopoietin-like protein 3 (ANGPTL3) is one of the major regulators for lipoprotein metabolism [21]. In 1999, ANGPTL3 was identified in human and mice and found highly conserved in both species [22–24]. It is specifically produced by hepatocytes in human [22, 25, 26] and then secreted into circulation. Numerous in vitro studies reported that the expression of ANGPTL3 is regulated by liver X receptor (LXR), insulin, angiopoietin-like protein 8 (ANGPTL8) [27, 28]. ANGPTL3 can be activated by the cleavage at proprotein convertase consensus site to release its N-terminal domain [29, 30]. Together with its family member angiopoietin-like protein 4 (ANGPTL4), they inhibit lipoprotein lipase activity including endothelial lipase activity and therefore, increase serum triglyceride and HDL-c [21, 31, 32]. Studies of Finnish population (n = 250) and American population (n = 1770) reported positive correlation between ANGPTL3 and HDL-c. However, ANGPTL3 level was associated with serum triglyceride in opposing directions in these two populations [33, 34]. Accordingly, ANGPTL3 deficient subjects are featured as reduced triglyceride, LDL-cholesterol (LDL-c) and HDL-c in the blood [35, 36].

Whereas all the studies so far relate ANGPTL3 to HDL-c in general population studies and in subjects with ANGPTL3 mutation, it is entirely unknown whether ANGPTL3 could be associated with HDL components and function rather than HDL-c. Except in vitro evidence, up to date, there is only one in vivo study showing insulin injection transiently reduced ANGPTL3 level in non-diabetic Caucasian women [25]. Thus, it remains unclear whether the negative regulation of insulin on ANGPTL3 could also exist in diabetic patients. To explore these questions, non-diabetic controls and type-2 diabetic patients were recruited in the study. Plasma levels of ANGPTL3 were quantified. The relationship between ANGPTL3 and HDL components or function was evaluated by a linear regression analysis adjusting for age, body mass index and lipid lowering drugs in male or female non-diabetic participants or type-2 diabetic patients, separately.

## Methods

### Human study

#### Study subjects and sample size

To explore the relationship between plasma level of ANGPTL3 with HDL components or function, non-diabetic participants and type-2 diabetic patients were randomly recruited from Lu He hospital in the analysis. The sample size was calculated by the standard formula (http://powerandsamplesize.com/Calculators/Compare-2-Means/2-Sample-1-Sided), in which 1-β is equal to 0.80 and α is equal to 0.01. According to the calculation, the sample size was 141. Therefore, 300 non-diabetic controls and 300 T2DM patients with clear diagnosis of T2DM were recruited from the Department of Endocrinology at Lu He hospital in Beijing. The criteria of T2DM diagnosis was adapted from that of WHO and ADA as following: (1) fasting glucose level ≥7 mmol/l; or (2) 2-h oral glucose tolerance test (OGTT) ≥11.1 mmol/l; or (3) random glucose level ≥11.1 mmol/l and accompanied by typical DM symptoms such as polydipsia, polyuria, increased food intake and loss of body weight. All the participants were Han people from the same region. They were free of liver and kidney disorders including carcinogenesis. General information including age, gender, disease history and medications was obtained. We excluded one non-diabetic male and one non-diabetic female because ANGPTL3 level or serum amyloid A value deviated more than 3 SDs from the mean.

The study complied with the Helsinki Declaration for investigation of human subjects. The entire study was obtained ethical approval from the competent Institutional Review Boards of Capital Medical University. All participants provided written informed consent.

#### Clinical measurement

Blood pressure was recorded by auscultation of the Korotkoff sounds, using a standard mercury sphygmomanometer. Blood pressure was the average of three readings. After overnight fasting, blood samples were obtained to measure total cholesterol, triglyceride, HDL-c, blood glucose, insulin and serum creatinine by the central laboratory in the hospital [37]. LDL-c was computed from serum total cholesterol and HDL-c and serum triglycerides by the Friedewald equation [38]. Body mass index was generated from body weight and height. Pancreatic β cell function and insulin resistance were computed by Homeostasis Model Assessment (Homa-β and HOMA–IR; http://www.dtu.ox.ac.uk/homacalculator/), using fasting insulin and glucose in the subjects. To assess kidney function, estimated glomerular filtration rate was calculated according to the formula [39].

#### ELISA for ANGPTL3

Plasma level of ANGPTL3 was quantified by ELISA, according to the instruction (IBL International GmBH, Japan) [24]. Intra- and interassay coefficients of variation were 5.9 and 6.8 % for ANGPTL3, respectively.

#### HDL isolation

Plasma samples (100 μl) were separated to obtain HDLs and LDL/VLDL fractions following the manual instruction (Biovision, CA, USA).

#### Quantification of HDL components

The concentrations of apoA-I, phospholipid, S1P and serum amyloid A in HDL fraction was measured according to the manual instructions, respectively (MLBio, Shanghai, China). Triglyceride concentration in HDL fractions were determined (BioSino Biotechnology, Beijing, China).

#### HDL preparation by ultracentrifugation

Plasma from human healthy participants and type-2 diabetic patients was separated by density gradient ultracentrifugation in a swing-out rotor described by Chapman et al. [40]. HDLs (1.063 g/ml < g < 1.21 g/ml) fractions were isolated, pooled and then dialyzed in 1 mM EDTA overnight. Cholesterol level was measured as above [41].

#### Cholesterol efflux assay

Cholesterol efflux assay was performed following the manual instruction (Biovision, CA, USA). In brief, RAW264.7 macrophages were plated at the density of 1 × 10^5^ cells/well in a 96-well plate and maintained in DMEM plus 10 % FBS (Sigma-Aldrich) for 2 h. The adherent cells were incubated with labeled cholesterol for 16 h and then exposed to 100 μg/ml HDLs for 4 h. The supernatant were transferred to a 96-well plate to measure the fluorescence (Ex/Em = 482/515 nm). The adherent cells were solubilized by cell lysis buffer to measure the fluorescence (Ex/Em = 482/515 nm). Cholesterol efflux % = fluorescence intensity of the media/(fluorescence intensity of the cell lysate + media) × 100 [42].

### Mice study

#### Mice and treatment

C57BL/6 and db/db mice at the age of 12 weeks old were used in the study. They were received intraperitoneal injection of insulin at 2 U/kg [43] and blood samples were collected to determine insulin and ANGPTL3 levels. The entire study was obtained ethical approval from the competent Institutional Review Boards of Capital Medical University.

#### Glucose tolerance test (IPGTT)

Mice were fasted for 8 h and then injected with 10 % glucose at 10 μl/g of body weight. Blood glucose was measured at 0, 15, 30, 60, 90 and 120 min before and after glucose administration.

#### ELISA for murine insulin and ANGPTL3

Plasma level of insulin and ANGPTL3 were measured by ELISA (Mercodia AB, Sweden and IBL International GmBH, Japan), respectively.

#### Hepatocyte isolation

Hepatocytes were isolated from age-matched db/db mice and age-matched C57BL/6 controls as described before [44]. Briefly, mice were anesthetized using 1.5 % pentobarbital sodium and a midline incision was made. Through the portal vein, the liver was perfused with Ca21/Mg21-free Honks’ Balanced Salt Solution containing glucose (10 mM) and HEPES (10 mM) at a flow rate of 3 ml/min for 10 min and then switched to liver digest medium containing collagenase P (Life Technologies, Inc) for another 10 min. After dissection, liver was minced into pieces and incubated in collagenase medium at 37 °C for 4 min for further digestion. The dissociated cells were dispersed by shaking followed by filtration through 100-mm nylon cell strainers (Becton–Dickinson, Franklin Lakes, NJ, USA). The cells were rinsed with 15 ml of low glucose Dulbecco’s modified Eagle’s medium (Sigma-Aldrich, St. Louis, MO, USA) containing 1 % bovine serum albumin, 0.8 mM oleate, 0.02 mg/ml dexamethasone, and 100 units of penicillin and 100 mg of streptomycin/ml and then collected by centrifugation at 1500 rpm/min for 5 min at 4 °C.

#### Cell culture and treatment

After isolation, hepatocytes were plated onto 60-mm mouse collagen IV-coated dishes (BioCoat, Becton–Dickinson) at a density of 1 × 10^6^ viable cells/dish in DMEM medium containing 10 % FBS [44]. They were allowed to attach overnight prior to experiment. After overnight serum deprivation, hepatocytes isolated from wide type mice were stimulated with 100 nM insulin or PBS for 10 min and Akt phosphorylation was assessed by western blot. To further confirm whether insulin could induce Akt phosphorylation in diabetic hepatocytes, after overnight serum deprivation, cells were incubated with or without 1 μM pAkt inhibitor VIII for 4 h and then stimulated with 100 nM insulin for 10 min.

To evaluate the effect of insulin on ANGPTL3 production, hepatocytes were serum starved for 24 h and then exposed to 100 nM insulin in the presence of absence of 1 μM Akt inhibitor VIII (Santa Cruz, CA, USA) for another 24 h. Cells were collected for western blot to study ANGPTL3 expression. In parallel, HepG2 were maintained in the same culture medium. After they reached 90 % confluency, they were serum starved and treated with 100 nM insulin as murine hepatocytes.

#### Western blot

Equal amount of protein lysates (40 μg) were separated on 8 % SDS-PAGE gel electrophoresis. After transfer to nitrocellulose membrane, the membrane was probed to polyclonal anti-mouse ANGPTL3 antibody (Abcam, Cambridge, UK), monoclonal rabbit anti-mouse phospho-Akt (Ser473), rabbit anti-mouse total Akt antibodies (Cell Signaling Technology, Beverly, MA, USA), and monoclonal anti-mouse GAPDH antibody (Santa Cruz, CA, USA). HRP-conjugated IgG secondary antibodies were purchased from Amersham Biosciences (Piscataway, NJ, USA). Western blot was quantified using the NIH image 1.62.

#### Statistics

Data are expressed as mean ± SD. Geometric data are expressed as mean with interquartile range or mean ± SD. For database management and statistical analysis in human study, we used the SAS system, version 9.3 (SAS Institute Inc., Cary, NC, USA). Serum triglyceride, fasting insulin in blood, Homa-β, Homa-IR and triglyceride content in HDL were logarithmically transferred to achieve normal distribution. Univariate analysis was performed between ANGPTL3 and each individual HDL component or the percentage of cholesterol efflux. Multivariate-adjusted analysis was performing by adjusting for age, body mass index and use of lipid lowering drugs as previously described [33, 34].

The covariables of ANGPTL3 in non-diabetic controls and T2DM patients were screened using a stepwise regression procedure with the p values for variables to enter and stay in the models set at 0.15. The covariables considered for non-diabetic controls and diabetic patients were age, body mass index, mean arterial pressure, lipid-lowering drugs (statins, niacin and fibrates) as one covariable, and classes of antihypertensive drugs [diuretics, vasodilators (α blockers and calcium channel blockers], and inhibitors of renin-angiotensin system [β–blockers, angiotensin-converting enzyme inhibitors and angiotensin receptor blockers)]. We standardized ANGPTL3 to the average in the population (mean or ratio) of significant covariables identified by stepwise regression and then regressed the standardized ANGPTL3 on insulin level in the circulation.

In both human and mice studies, unpaired, 2-tailed Student’s test and Fisher’s exact test were used to compare the means and the proportions between non-diabetic subjects and T2DM patients, respectively. For more than two experimental groups, one-way analysis of variance (ANOVA) with Dunnett Multiple Comparison test was applied. Significance was a two-tailed α-level of 0.05 or less.

## Results

### General characterisation of the study subjects

Equal amount of males and females were enrolled in non-diabetic and type-2 diabetic groups in the study. After exclusion of the outliers, 298 non-diabetic controls and 300 diabetic patients were analyzed. Among female subjects, in 149 non-diabetic controls (50.0 % women), age averaged (SD) 52.9 (8.2) years, body mass index 26.2 (3.9) kg/m^2^, and blood pressure 131.8 (20.8) mm Hg systolic and 76.8 (10.6) mm Hg diastolic. Mean values were 54.6 (12.8) mg/dL for HDL-c and 131.4 (28.6) mL/min/1.73 m^2^ for eGFR. In 150 female T2DM patients (50.0 % women), age averaged (SD) 56.6 (6.0) years, body mass index 26.4 (4.0) kg/m^2^, and blood pressure 138.8 (17.8) mm Hg systolic and 77.1 (11.2) mm Hg diastolic. Mean values were 49.8 (9.7) for HDL-c and 116.8 (32.9) mL/min/1.73 m^2^ for eGFR. In male subjects, body mass index increased from 25.2 (4.1) kg/m^2^ in non-diabetic participants to 26.5 (3.4) kg/m^2^ in diabetic patients (p = 0.004). eGFR was 118.1 ml/min/1.73m2 in non-diabetic males and decreased to 105.6 ml/min/1.73m2 in male diabetic patients (p = 0.002). HDL-c was 52.9 (13.8) mg/dL in non-diabetic subjects and 43.8 (13.9) mg/dL in diabetic patients (p < 0.0001). Table 1 lists general characteristics of non-diabetic and T2DM patients by gender.

**Table 1:** General characteristics of male and female T2DM patients and non-diabetic subjects

Gender/characteristic	Female		Male	
	Non-diabetic	T2DM	Non-diabetic	T2DM
Number of females	149	150	149	150
N in category, (%)				
Hypertension	47 (31.5 %)	63 (42.0 %)	36 (24.5 %)	69 (46.0 %)^ł^
Cardiovascular disease	6 (4.0 %)	14 (9.3 %)	8 (5.4 %)	22 (14.7 %)^ł^
Anti-hypertensive Medications				
Diuretics	2 (1.3 %)	2 (1.3 %)	2 (1.3 %)	7 (4.7 %)
β blocker	4 (2.7 %)	12 (8.0 %)*	3 (2.0 %)	7 (4.7 %)
Calcium channel blocker	7 (4.7 %)	24 (16.0 %)	9 (6.0 %)	32 (21.3 %)^ǂ^
α blocker	4 (2.7 %)	11 (7.3 %)	0 (0 %)	17 (11.3 %)^§^
ACE inhibitors/ARB	3 (2.0 %)	7 (4.7 %)	2 (1.3 %)	14 (9.3 %)^ǂ^
Lipid lowering treatment	4 (2.7 %)	2 (1.3 %)	4 (2.7 %)	22 (14.7 %)^ǂ^
Anti-diabetic treatment				
Insulin	NA	20 (13.3 %)	NA	22 (14.7 %)
Sulfonylureas	NA	61 (40.7 %)	NA	32 (21.3 %)
Metformin	NA	94 (62.7 %)	NA	62 (41.3 %)
α glycosidase inhibitors	NA	70 (46.7 %)	NA	42 (28.0 %)
Mean (SD)				
Age (years)	52.9 (8.2)	56.6 (6.0)^§^	53.8 (8.3)	54.5 (10.9)
Body mass index (kg/m^2^)	26.2 (3.9)	26.4 (4.0)	25.2 (4.1)	26.5 (3.4)^ł^
Systolic pressure (mm Hg)	131.8 (20.8)	138.8 (17.8)^ł^	133.8 (18.9)	135.2 (19.8)
Diastolic pressure (mm Hg)	76.8 (10.6)	77.1 (11.2)	78.9 (12.1)	82.7 (10.8)^ǂ^
eGFR (ml/min/1.73 m^2^)	131.4 (28.6)	116.8 (32.9)^§^	118.1 (28.3)	105.6 (42.5)^ł^
Total cholesterol (mg/dL)	195.9 (35.4)	195.1 (35.8)	187.0 (29.3)	176.8 (40.0)*
LDL cholesterol (mg/dL)	118.3 (35.7)	121.4 (32.2)	111.3 (24.8)	108.5 (34.0)
HDL cholesterol (mg/dL)	54.6 (12.8)	49.8 (9.7)^ǂ^	52.9 (13.8)	43.8 (13.9)^§^
Fasting blood glucose (mmol/L)	5.0 (0.4)	8.3 (2.4)^§^	5.1 (1.5)	8.9 (2.8)^§^
Geometric mean (IQR)				
Triglyceride (mg/dL)	111.5 (81.4–176.1)	123.7 (117.8–129.8)	106.0 (100.3–112.0)	150.8 (140.4–161.9)^§^
Insulin (pmol/L)	50.9 (37.2–71.4)	58.9 (56.2–63.1)*	38.5 (36.4–40.7)	33.6 (30.3–37.2)
Homa-β	92.0 (76.2–110.8)	41.7 (38.9–43.7)^§^	75.5 (71.9–79.4)	24.1 (22.0–26.4)^§^
Homa-IR	0.97 (0.69–1.32)	1.25 (0.80–1.91)^§^	0.77 (0.47–1.14)	0.78 (0.29–1.50)

*eGFR* estimated glomerular filtration rate; *ACE* inhibitors Angiotensin-converting enzyme inhibitors; *ARB* angiotensin receptor blocker; *LDL* low-density lipoprotein; *HDL* high-density lipoprotein; Homa-βand Homa-IR were computed by Homeostasis Model Assessment algorithm (http://www.dtu.ox.ac.uk/homacalculator/) using fasting insulin and fasting blood glucose; *IQR* interquartile range

* p ≤ 0.05; ^ł^ p ≤ 0.01; ^ǂ^ p ≤ 0.001; and ^§^ p ≤ 0.0001 when compared with sex-matched controls

Plasma samples were fractionated to isolate HDLs and the concentrations of apoA-I, serum amyloid A, phospholipid, triglyceride and sphingosine-1-phosphate in HDLs were determined. Both male and female T2DM patients were featured as decrease of apoA-I concentration but increase of serum amyloid A and phospholipid levels in HDLs compared with non-diabetic controls (Table 2).

**Table 2:** Comparison of HDL components in non-diabetic subjects and T2DM patients

Characteristics	All		Female		Male	
	Non-diabetic	T2DM	Non-diabetic	T2DM	Non-diabetic	T2DM
N in category	298	300	149	150	149	150
Cholesterol (mg/dL)	53.8 (13.3)	46.8 (12.3)^§^	54.6 (12.8)	49.8 (9.7)^**ǂ**^	52.9 (13.88)	43.8 (13.9)^§^
ApoA-I (μg/mL)	7.8 (2.2)	6.8 (2.6)^§^	7.5 (2.0)	6.9 (2.6)^ł^	8.0 (2.4)	6.9 (2.6)^**ǂ**^
Serum amyloid A (μg/L)	811.9 (286.8)	928.5 (326.8)^§^	924.8 (265.3)	1021.9 (335.2)*	691.6 (244.4)	836.0 (291.0)^§^
Phospholipid (pg/mL)	963.8 (438.4)	1080.5 (391.1)^**ǂ**^	992.4 (413.8)	1045.9 (349.6)	933.6 (462.3)	1115.1 (427.0)^**ǂ**^
sphingosine-1-phosphate (nmol/L)	1549.7 (473.3)	1464.3 (699.9)	1465.0 (450.5)	1429.4 (769.2)	1629.3 (480.2)	1499.3 (623.6)*
Triglycerides (log, mg/dl)	13.6 (10.8–16.4)	13.0 (10.6–17.6)	12.3 (9.7–16.0)	15.0 (11.4–19.4)^**ǂ**^	14.0 (11.7–16.8)	11.8 (9.4–15.0)

^§^ p < 0.0001;^ǂ^ p < 0.001; ^ł^ p < 0.01; * p < 0.05 when compared with non-diabetic controls

### Plasma levels of ANGPTL3 in the entire study subjects

**Fig. 1:**
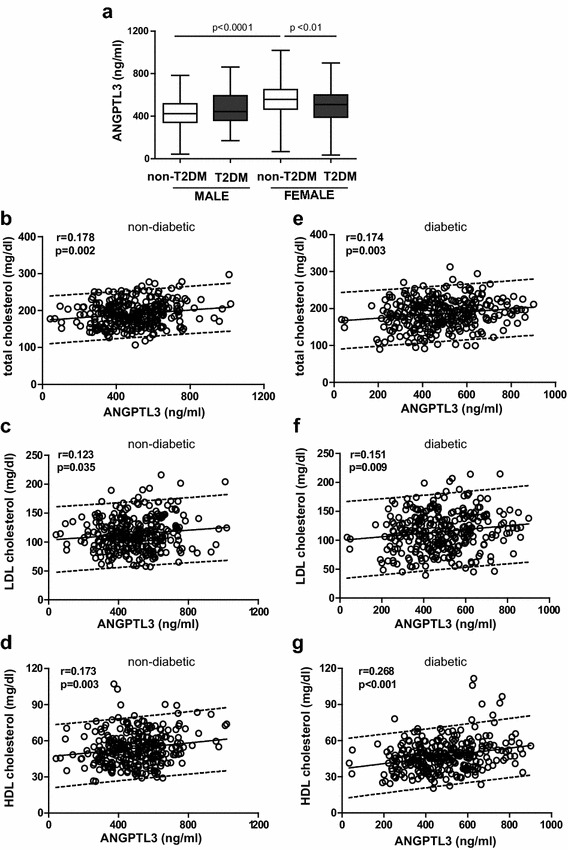
Plasma levels of ANGPTL3 and its association with total cholesterol, LDL-c or HDL-c in non-diabetic subjects and T2DM patients. **a** Quantification of plasma level of ANGPTL3 in non-diabetic subjects and T2DM patients. Univariate analysis showed the correlation between ANGPTL3 and total cholesterol (**b**), LDL-c (**c**) and HDL-c (**d**) in non-diabetic controls (n = 299). In parallel, the correlation between ANGPTL3 and total cholesterol (**e**), LDL-c (**f**) and HDL-c (**g**) in T2DM patients (n = 300). The *parallel lines* defined the 90 % prediction band

Quantification of ANGPTL3 revealed that plasma level of ANGPTL3 did not differ between non-diabetic controls and T2DM patients (493.5 ± 162.4 vs. 482.9 ± 160.1 ng/ml, p = 0.42, n = 298–300). However, plasma level of ANGPTL3 was greater in females than males in non-diabetic groups (558.6 ± 164.6 vs. 428.3 ± 131.6 ng/ml, p < 0.0001) (Fig. 1a). Although ANGPTL3 level was similar between male non-diabetic and T2DM subjects (428.3 ± 131.6 vs. 467.2 ± 159.3 ng/ml, p = 0.10), ANGPTL3 level was significantly reduced in female T2DM patients compared with female non-diabetic participants (558.6 ± 164.6 vs. 498.5 ± 159.9 ng/ml, p < 0.01) (Fig. 1a). Distribution and probability plots of ANGPTL3 level in both non-diabetic controls and diabetic patients are shown in Additional file 1: Figure S1.

### Unadjusted analysis

In line with previous reports [34], univariate analysis showed that plasma level of ANGPTL3 was positively correlated with total cholesterol, LDL-c and HDL-c in non-diabetic controls (Fig. 1b–d). Similarly, positive association was found between ANGPTL3 levels and total cholesterol, LDL-c and HDL-c in T2DM patients (Fig. 1e–g). Nevertheless, no significant association was detected between ANGPTL3 and serum triglyceride in both groups (non-diabetic: r = 0.035, p = 0.542; T2DM: r = −0.058, p = 0.313).

In female controls, the positive association between ANGPTL3 level and total cholesterol or HDL-c remained but not with LDL-c (total cholesterol: r = 0.188, p = 0.027; LDL-c: r = 0.140, p = 0.088; HDL-c: r = 0.189, p = 0.021). Slightly different from female controls, ANGPTL3 levels was correlated only with HDL-c in female T2DM patients (total cholesterol: r = 0.158, p = 0.054; LDL-c: r = 0.139, p = 0.091; HDL-c: r = 0.186, p = 0.022). Different from females, a positive correlation between ANGPTL3 level and HDL-c was observed in male diabetic patients (r = 0.307, p = 0.0001) not in male controls (r = 0.153, p = 0.089) (Table 3).

**Table 3:** Univariate analysis of correlation between angptl3 and lipids or HDL components in the study subjects

Controls vs. T2DM	Female controls		Female T2DM		Male controls		Male T2DM	
	r	p	r	p	r	p	r	p
N in category	149		150		149		150	
Total cholesterol	0.188	0.027	0.158	0.054	0.093	0.273	0.155	0.058
Triglyceride	0.027	0.746	0.011	0.889	−0.056	0.518	−0.084	0.607
LDL cholesterol	0.140	0.088	0.139	0.091	0.040	0.628	0.133	0.106
HDL components								
Cholesterol	0.189	0.021	0.186	0.022	0.153	0.089	0.307	0.0001
ApoA-I	0.587	<0.0001	0.477	<0.0001	−0.015	0.889	0.119	0.146
Serum amyloid A	−0.209	0.011	0.051	0.539	−0.058	0.471	−0.044	0.594
Triglyceride (log)	0.018	0.832	0.012	0.881	0.123	0.141	0.185	0.023
Phospholipid	−0.004	0.959	−0.108	0.190	−0.131	0.113	0.179	0.028
Sphingosine-1-phosphate	−0.014	0.869	0.163	0.046	−0.060	0.473	0.009	0.908

**Fig. 2:**
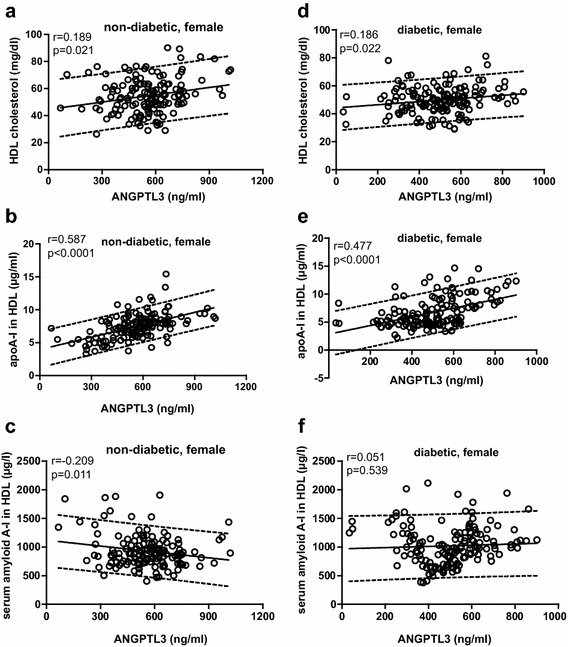
Univariate association between plasma level of Angptl3 and HDL-c, apoA-I or serum amyloid A in non-diabetic females (**a**–**c**) and diabetic females (**d**–**f**). The parallel lines defined the 90 % prediction band. Significance: ^§^p < 0.0001; ^ǂ^p < 0.001; ^ł^p < 0.01; * p < 0.05

When looking at the association between ANGPTL3 and other HDL components, ANGPTL3 was highly positively associated with apoA-I but negatively associated with SAA in HDLs of non-diabetic female subjects (apoA-I: r = 0.587, p < 0.0001; SAA: r = −0.209, p = 0.028). Differently, ANGPTL3 stayed positively associated with apoA-I and S1P in HDLs of female T2DM patients (apoA-I: r = 0.477, p < 0.0001; S1P: r = 0.163, p = 0.046) but not with SAA (r = 0.051, p = 0.539). Distinct from female controls, ANGPTL3 levels did not correlate with HDL components in non-diabetic male subjects. Table 3 summarizes the univariate analysis of correlation between ANGPTL3 and lipids or HDL components between male and female non-diabetic controls and T2DM patients. Univariate analysis of the associations between ANGPTL3 and HDL-c, apoA-I or serum amyloid A in female non-diabetic participants and diabetic patients were shown in Fig. 2a–f.

### Multivariable-adjusted analysis

Thereafter, we performed a linear regression analysis between ANGPTL3 and individual HDL component by adjusting for age, body mass index and use of Statin and Niacin. In female non-diabetic patients, ANGPTL3 level was positively correlated with HDL-c and apoA-I but inversely correlated with SAA. Per 1-SD increment of ANGPTL3, the changes amounted to +2.57 mg/dL HDL-c (p = 0.017), +1.14 μg/mL apoA-I (p < 0.0001) and −47.07 μg/L SAA (p = 0.032). In female diabetic patients, the corresponding estimates were 1.69 mg/dL HDL-c (p = 0.035), +1.25 μg/mL apoA-I (p < 0.0001) and −11.71 μg/L SAA (p = 0.676) (Table 4).

**Table 4:** Multivariate analysis of correlation between angptl3 and HDL components in the study subjects

Controls vs. T2DM	Female controls	Female T2DM	Male controls	Male T2DM
	Estimate (95 % CI)	Estimate (95 % CI)	Estimate (95 % CI)	Estimate (95 % CI)p
N in category	149	150	149	150
HDL components				
Cholesterol (mg/dL)	2.57 (0.48 to 4.65)*	1.69 (0.13 to 3.25)*	2.07 (0.14 to 4.00)*	4.36 (2.08 to 6.64)^ł^
ApoA-I (μg/mL)	1.14 (0.88 to 1.40)^§^	1.25 (0.87 to 1.63)^§^	−0.03 (−0.42, 0.36)	0.38 (−0.02 to 0.78)
Serum amyloid A(μg/L)	−47.07 (−89.60 to −4.53)*	−11.7 (−43.15 to 66.57)	−14.19 (−53.93 to 25.56)	−6.16 (−50.99 to 38.66)
Triglyceride (log, mg/dL)	1.00 (0.93 to 1.08)	1.00 (0.93 to 1.07)	1.03 (0.97 to 1.09)	1.12 (1.04 to 1.20)*
Phospholipid (pg/mL)	−5.96 (−74.81 to 62.89)	−37.24 (−91.14 to 16.66)	−54.71 (−127.59 to 18.18)	83.09 (15.18 to 151.00)*
Sphingosine-1-phosphate (nmol/L)	−14.33 (−88.89 to 60.24)	−124.3 (−0.64 to 249.30)	−28.02 (−106.40 to 50.37)	16.94 (−84.79 to 118.67)

All associations were adjusted for age, body mass index and lipid lowering drugs (Statins and Niacin). Estimates given with 95 % CI express the difference in HDL components associated with 1-SD increase of ANGPTL3

Significance: ^§^ p < 0.0001; ^ł^ p < 0.01; * p < 0.05

Similar as univariate association results, after adjusting for age, body mass index and lipid lowering drugs, ANGPTL3 was only associated with HDL-c in male controls (Table 4). No significant correlation was seen between ANGPTL3 and apoA-I in male controls and T2DM patients (non-diabetic: p = 0.91; T2DM: p = 0.07). Nevertheless, positive associations between ANGPTL3 and triglyceride or phospholipid in HDL were detected in male T2DM patients (Table 4).

Put together, ANGPTL3 levels were intimately associated with HDL-c, apoA-I and SAA in females non-diabetic controls.

### Correlation between ANGPTL3 levels and HDL function

As described earlier, the beneficial atheroprotective properties of HDL are exerted by the components in HDL particles, in which apoA-I improves but SAA adversely impairs HDL function. After we observed the diverse association between ANGPTL3 and SAA in HDLs in females non-diabetic and diabetic subjects, we further dissected the relationship between ANGPTL3 and HDL function. RAW264.7 macrophages were preloaded with fluorescently labeled cholesterol and then exposed to 100 μg/ml HDLs isolated from female non-diabetic controls or T2DM patients. The percentage of cholesterol efflux was determined 4 h after addition of HDL.

**Fig. 3:**
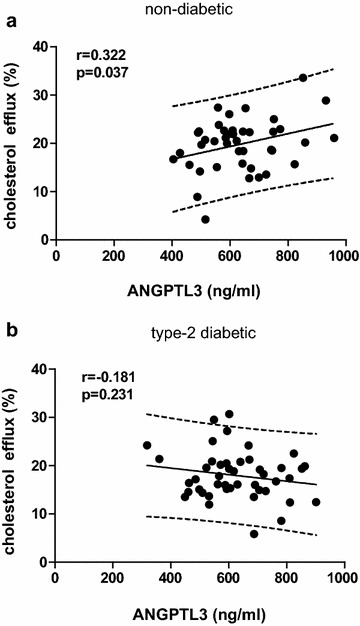
Association between ANGPTL3 levels and HDL function in non-diabetic and T2DM female subjects. HDL was isolated from plasma of non-diabetic or T2DM female subjects. RAW264.7 macrophages were loaded with fluorescent labeled cholesterol for 16 h and then exposed to 100 μg/ml HDLs for 4 h. Fluorescence intensity in the supernatant and cells were determined to calculate the percentage of cholesterol efflux towards HDLs. The correlation between ANGPTL3 levels and the percentage of cholesterol efflux in female non-diabetic participants (n = 42) or T2DM patients (n = 45) is shown in **a** and **b**, respectively. The *parallel lines* defined the 90 % prediction band

Univariate analysis illustrated that plasma levels of ANGPTL3 was significantly positively associated with the percentage of cholesterol efflux in non-diabetic controls but not in female T2DM patients (non-diabetic: r = 0.322, p = 0.037, n = 42; T2DM: r = −0.181, p = 0.231, n = 45) (Fig. 3a, b). After adjusting for age, body mass index and use of lipid lowering drugs, per 1-SD increase of ANGPTL3, the estimate was 2.11 % in non-diabetic controls but weakened to −1.46 % in female T2DM patients (non-diabetic: p = 0.07; T2DM: p = 0.13).

**Fig. 4:**
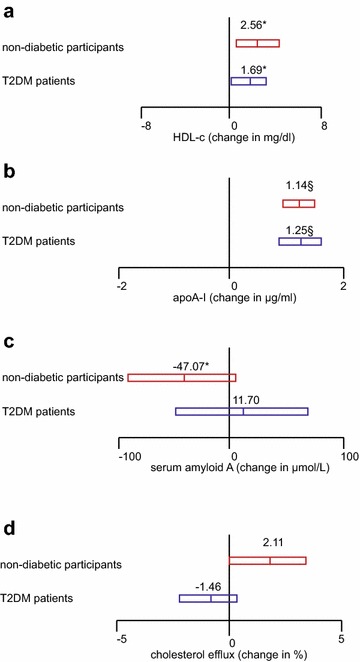
Plasma level of ANGPTL3 in relationship to HDL components and function in non-diabetic female subjects and female T2DM patients. Regression was performed between standardized ANGPTL3 and cholesterol (**a**), apoA-I (**b**), Serum Amyloid A (**c**) and the percentage of cholesterol efflux (**d**) in HDLs in non-diabetic female subjects and female T2DM patients. Estimates express as change in unit with 1-SD increase of ANGPTL3 in non-diabetic subjects and T2DM patients, respectively. ^§^p < 0.0001; ^ǂ^p < 0.001; ^ł^p < 0.01; * p < 0.05

Taken together, one SD increase of ANGPTL3 (161.8 ng/ml) associated with increase of 2.56 mg/dL cholesterol (95 % CI 0.48, 4.65; p = 0.017), 1.14 μg/mL apoA-I (95 % CI 0.88, 1.40; p < 0.0001) and decrease of 47.07 μg/L SAA in HDL particles in non-diabetic female subjects (95 % CI −89.60, −4.53; p = 0.032) (Fig. 4a–c). By contrast, 1-SD increase of ANGPTL3 (136.1 ng/ml) associated with increase of 1.69 mg/dl cholesterol (95 % CI 0.13, 3.25, p = 0.037), 1.25 μg/mL apoA-I (95 % CI 0.88, 1.63; p < 0.0001) and decrease of 11.70 μg/L SAA (95 % CI −43.15, 66.57; p = 0.676) in HDLs of female T2DM patients (Fig. 4a–c). When translating into HDL function, one SD increase of ANGPTL3 related to increase of 2.11 % cholesterol efflux against HDLs in non-diabetic females (95 % CI −0.11, 4.33, p = 0.071) but decrease of 1.46 % in female T2DM patients (95 % CI −3.31, 0.39; p = 0.130) (Fig. 4d).

### The effect of insulin treatment on ANGPTL3 level in diabetic patients and mice

Finally, we screened covariables that could affect ANGPTL3 level by stepwise regression in non-diabetic participants or T2DM patients. Recent studies showed that insulin decreased ANGPTL3 level in HepG2 cells in vitro [45] and infusion of insulin to non-diabetic Caucasian women (n = 18) transiently reduced ANGPTL3 level in the blood [25]. Therefore, the covariables that were included in the model were age, sex, mean arterial pressure, body mass index, plasma insulin level, classes of anti-hypertensive medications, and lipid-lowering drugs. Additional file 1: Table S1 lists the identified covariables that influenced ANGPTL3 levels.

**Fig. 5:**
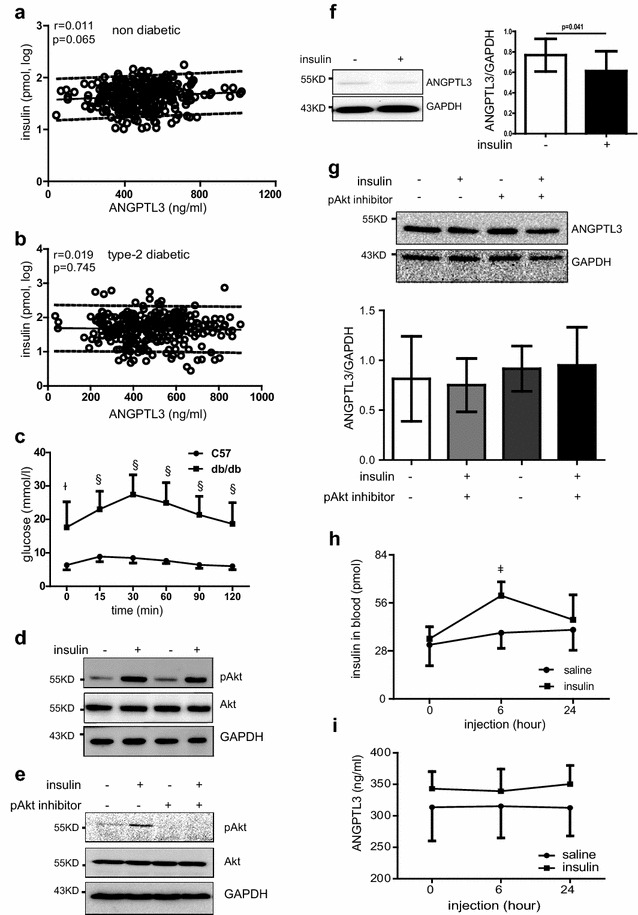
The effect of insulin on ANGPTL3 level in diabetic subjects. Unadjusted correlation analysis between plasma insulin level and ANGPTL3 level in non-diabetic participants (**a**) and diabetic patients (**b**). The parallel lines defined the 90 % prediction band. C57BL/6 and db/db mice were received peritoneal injection of 10 % glucose at 10 μl/g of body weight. Blood glucose was determined (**c**). **d** Representative western blot of pAkt and Akt in normal hepatocytes stimulated with 100 nM insulin ex vivo. (E) Western blot showing the expression of pAkt and Akt in diabetic hepatocytes stimulated with 100 nM insulin in the presence or absence of 1 μM pAkt inhibitor. **f** Hepatocytes were isolated from diabetic db/db mice and stimulated with 100 nM insulin in the presence or absence of pAkt inhibitor VIII. Representative western blot of pAkt and totak Akt in diabetic hepatocytes ex vivo. **f** ANGPTL3 and GAPDH expression in HepG2 cells by western blot and quantification of ANGPTL3 expression when adjusted by GAPDH expression. **g** Hepatocytes isolated from db/db mice were treated with 100 nM insulin in the presence or absence of Akt inhibitor VIII. Representative western blot of ANGPTL3 and GAPDH. ANGPTL3 expression was quantified by adjusting with GAPDH expression. Db/db mice were injected with insulin 2 U/kg. Plasma insulin (**h**) and ANGPTL3 was quantified (**i**). ^§^p < 0.0001; ^ǂ^p < 0.001; ^ł^p < 0.01

Univariate analysis elucidated that plasma insulin level did not relate to ANGPTL3 level in male and female diabetic patients (Fig. 5a, b). Next, we performed stepwise regression for covariables of ANGPTL3. The identified covariables that influenced ANGPTL3 were summarized in Additional file 1: Table S1. We standardized ANGPTL3 to the average (mean or proportion) of the aforementioned covariables identified by stepwise regression in the two study populations. No significant relationship was detected between standardized ANGPTL3 and insulin in both populations (p > 0.75).

To further testify the effect of insulin on ANGPTL3 level, hepatocytes from in db/db mice were isolated from db/db mice and their age-matched controls. At the age of 12-week old, db/db mice developed typical impaired glucose tolerance after challenge by intraperitoneal injection of glucose (Fig. 5c). After isolation and overnight fasting, hepatocytes were stimulated with 100 nM insulin for 10 min and Akt phosphorylation was seen induced 2.5- and 1.7-fold in both normal and diabetic hepatocytes, respectively (Fig. 5d, e, n = 3–4). Consistent with previous reports [25, 45], western blot experiments illustrated that insulin decreased ANGPTL3 production in human hepatoma HepG2 cells (Fig. 5f). As numerous studies showed PI3 K/Akt physphorylation are key molecules downstream of hepatic insulin signaling pathways [46, 47], hepatocytes of db/db mice were treated with 100 nM insulin with or without pAkt inhibitor for 24 h to evaluate ANGPTL3 production. Different from HepG2 cells, neither insulin treatment nor pAkt inhibitor altered ANGPTL3 production in diabetic hepatocytes in vitro (Fig. 5g). Likewise, injection of insulin to db/db mice did not lead to any change of ANGPTL3 level in the circulation (Fig. 5h, i).

## Discussion

The key findings in the study include: (1) ANGPTL3 level was reduced in female T2DM patients compared with female non-diabetic subjects whereas ANGPTL3 level was similar between male non-diabetic and T2DM subjects; (2) after adjusting for the confounding factors, ANGPTL3 levels were positively correlated with the concentrations of cholesterol and apoA-I but negatively correlated with SAA in HDLs in non-diabetic female subjects. However, ANGPTL3 was positively associated with HDL-c and apoA-I but not with SAA in HDLs in female diabetic patients. (3) ANGPTL3 level was positively correlated with HDL function in the aspect of cholesterol efflux against HDLs in female controls but weakened in female T2DM patients; (4) plasma insulin level had no correlation with ANGPTL3 levels in diabetic patients.

### ANGPTL3, HDL components and HDL function

Accumulating evidence has documented the positive correlation between ANGPTL3 and total cholesterol or HDL-c in different populations [33, 34], our findings in the entire non-diabetic participants are consistent with these findings. However, when we further dissected the relationship by genders, we found that ANGPTL3 levels were positively correlated with HDL-c but not with total cholesterol in female non-diabetic subjects. Surprisingly, ANGPTL3 was positively associated with total cholesterol but not with HDL-c in male non-diabetic subjects. Further investigation is needed to explore the mechanisms underlying the diverse relationship between ANGPTL3 and HDL-c in males and females. Nonetheless, these data strongly pointed out the specific relationship between ANGPTL3 and HDLs in females.

After adjusting for cofounding factors, ANGPTL3 was highly positively associated with HDL-c and apoA-I in female non-diabetic participants and diabetic patients. On top of that, negative correlation between ANGPTL3 and SAA in HDL was observed in non-diabetic females but it was abrogated in female patients. To be noted, the regression coefficient between ANGPTL3 and SAA was significantly higher in female non-diabetic controls than female T2DM patients (−0.286 vs. 0.073, p = 0.002). Consequently, ANGPTL3 levels were found positively associated with the percentage of cholesterol efflux towards HDLs. These findings suggest the substantial relationship of ANGPTL3 on HDL components mainly HDL-c, apoA-I, and SAA and HDL function in female non-diabetic subjects.

Previous studies have shown that ANGPTL3 regulates HDL cholesterol through suppression of endothelial lipase (EL) [31, 32], which is an enzyme to hydrolyze HDL cholesterol and accelerate its catabolism. In EL-deficient mice, plasma level of HDL cholesterol and apoA-I were both increased, resulting in enhanced cholesterol efflux [48]. The increased apoA-I expression in EL-deficient mice could come from post-transcriptional regulatory mechanism because apoA-I production in hepatocytes was comparable between EL-deficient mice and controls [49]. In line with these findings, inhibition of proprotein convertases by profurin increases EL activity. In Ad-profurin-treated mice, reduced HDL-cholesterol and apoA-I level were observed which was accompanied with reduced cholesterol efflux. Although profurin expression had no effect on apoA-I excretion, the mature form of apoA-I was seen largely reduced in hepatocytes of Ad-profurin-treated mice [31]. These data suggest the effect of endothelial lipase on posttranslational modification of apoA-I.

In our study, we demonstrated that ANGPTL3 level was significantly lower in female diabetic patients than non-diabetic controls (558.6 ± 164.6 vs. 428.3 ± 159.3 ng/mL, p < 0.0001). HDL-c and apoA-I concentration were both reduced in female diabetic patients compared to female controls (7.8 ± 2.2 vs. 6.8 ± 2.6 μg/dL, p < 0.0001). We observed negative correlation between ANGPTL3 and SAA in HDL in non-diabetic females but not in female patients. In addition, the regression coefficient between ANGPTL3 and SAA was significantly higher in female non-diabetic controls than female T2DM patients (−0.286 vs. 0.073, p = 0.002). It has been shown that SAA affects the composition of HDL via releasing apoA-I from HDL [50, 51]. Taken together, in the context of diabetes, we speculate that reduced ANGPTL3 in female diabetic patients might jeopardize its inhibitory effect on EL, weaken its association with SAA and adversely affect apoA-I stability, all of which would contribute to reduced apoA-I level and HDL dysfunction. Consequently, the association between ANGPTL3 and cholesterol efflux became weaker in female diabetic patients. Nevertheless, how ANGPTL3 interplays with apoA-1 and SAA for HDL metabolism and function needs to be further investigated.

### Insulin and ANGPTL3

Consistent with previous report, we found that ANGPTL3 levels were much lower in male non-diabetic participants than female controls in the study [33]. When we screened covariables by stepwise regression, different covariables were identified in males or females in the presence or absence of T2DM. These data implicated different regulatory mechanisms of ANGPTL3 levels in males and females.

Insulin therapy is a crucial tool in treating diabetic patients. It is not only beneficial for glycemic control but also improve complications that are secondary to hyperglycemia especially in elder patients [52]. Previous studies revealed the down-regulation of ANGPTL3 production in immortalized human hepatocytes by insulin [25, 45, 53]. Likewise, insulin injection transiently decreased ANGPTL3 level in the blood of non-diabetic Caucasian women with small sample size. In contrast to these findings, the data of our study clarified no effect of insulin on ANGPTL3 levels by several lines of evidence: (1) neither univariate nor multivariate analysis showed a significant correlation between plasma insulin level and ANGPTL3 in the study subjects; (2) insulin treatment did not change ANGPTL3 production in hepatocytes isolated from diabetic db/db mice; and (3) ANGPTL3 level did not alter after 6 and 24 h injection of insulin. These data collectively suggested the complicated regulation of ANGPTL3 in the setting of diabetes. Giving that the previous in vivo study was small (n = 18) and confined to selected healthy volunteers, the effect of insulin on ANGPTL3 level needs further confirmation from larger sample sizes with different ethnic groups.

### Study limitations

There are limitations in the study. First, ANGPTL3 level could be also influenced by other factors. Recently, another family member, ANGPTL8 was identified, which might regulate ANGPTL3 activity [28]. ANGPTL8, also named as Betatrophin, was originally identified to induce β cell proliferation in mice. A recent study has reported a strong association between angptl8 and C-peptide level in non-diabetic subjects, highlighting the likelihood that this increase of ANGPTL8 driven by insulin resistance serves to compensate for the increased insulin demand in obese subjects [54]. Except its role in C-peptide production, ANGPTL8 also regulates lipid metabolism. GWAS studies in humans show ANGPTL8 SNPs affect HDL-c or LDL-c but the effect of ANGPTL8 on triglyceride is modest. Microarray analysis reveals that ANGPTL8 ANGPTL3, ANGPTL4 coordinates with ANGPTL3 and ANGPTL4 in the regulation of lipids [55]. Thus, it would be interesting to dissect whether ANGPTL8 acts as a novel independent variable in the association between ANGPTL3 and HDL components. Second, due to technical limitations, it is not feasible to evaluate ANGPTL3 activity in vivo. Third, we investigated the relationship between ANGPTL3 and major HDL components. As the progress of high-through proteomics, new molecules would be identified in HDL particles. Further evaluation of ANGPTL3 on HDL particles will be continued.

## Conclusion

ANGPTL3 is highly positively correlated with cholesterol and apoA-I but negatively correlated with SAA in HDL in non-diabetic female subjects. Paradoxically, plasma levels ANGPTL3 was reduced in female T2DM patients and its negative association with SAA was diminished. In line with that, plasma levels of ANGPTL3 were positively correlated with the percentage of cholesterol efflux towards HDL in non-diabetic females but not in female T2DM patients. Therefore, ANGPTL3 could be specifically associated with HDL metabolism and function in females. The decrease of ANGPTL3 level in female diabetes patients might be involved in the impaired HDL metabolism and function.

## Additional file

10.1186/s12933-016-0450-1 Additional figure and table.

## Abbreviations

ANGPTL3: angiopoietin-like protein 3
HDL-c: high-density lipoprotein-cholesterol
T2DM: type 2 diabetes mellitus
rHDL: reconstituted HDL
apoA-I: apolipoprotein A-I
SAA: serum amyloid A
S1P: sphingosine-1-phosphate
LXR: liver X receptor
ANGPTL8: angiopoietin-like protein 8
ANGPTL4: angiopoietin-like protein 4
LDL-c: low-density lipoprotein-cholesterol
OGTT: oral glucose tolerance test
IPGTT: glucose tolerance test
eGFR: estimated glomerular filtration rate
ANOVA: one-way analysis of variance
