# Supplementary material for: Different relationship between ANGPTL3 and HDL components in female non-diabetic subjects and type-2 diabetic patients
Source: Cardiovasc Diabetol. 2016 Sep 13;15(1):132. doi: 10.1186/s12933-016-0450-1 (PMC5020513; doi:10.1186/s12933-016-0450-1)
Supplement: Supplementary file 1 — 10.1186/s12933-016-0450-1 Additional figure and table. [file 12933_2016_450_MOESM1_ESM.docx]

**Supplemental Materials**

Different relationship between ANGPTL3 and HDL components in non-diabetic subjects and type-2 diabetic patients

Running title: ANGPTL3 and HDL

Dong Zhao,^1§^ Long-Yan Yang,^1§^ Xu-Hong Wang, ^1^ Sha-Sha Yuan, ^1^ Cai-Guo Yu, ^1^Zong-Wei Wang, ^1^Jia-Nan Lang, ^1^,Ying-Mei Feng^1,2^

Affiliations:

1. Beijing Key laboratory of Diabetes Prevention and Research, Department of Endocrinology, Lu He hospital, Capital Medical University, Beijing, China

2. Stem Cell Institute, University of Leuven, Leuven, Belgium

§ Both authors share equal contribution to the study.

Correspondence to:

Ying-Mei Feng MD, Ph.D

1. Beijing Key laboratory of Diabetes Prevention and Research, Department of Endocrinology, Lu He hospital, Capital Medical University, Beijing 101149, China

2. Stem Cell Institute, University of Leuven, Leuven 3000, Belgium

E-mail address: yingmeif13@sina.com

Tel: 0086-10-69543901

Fax: 0086-10-69531069


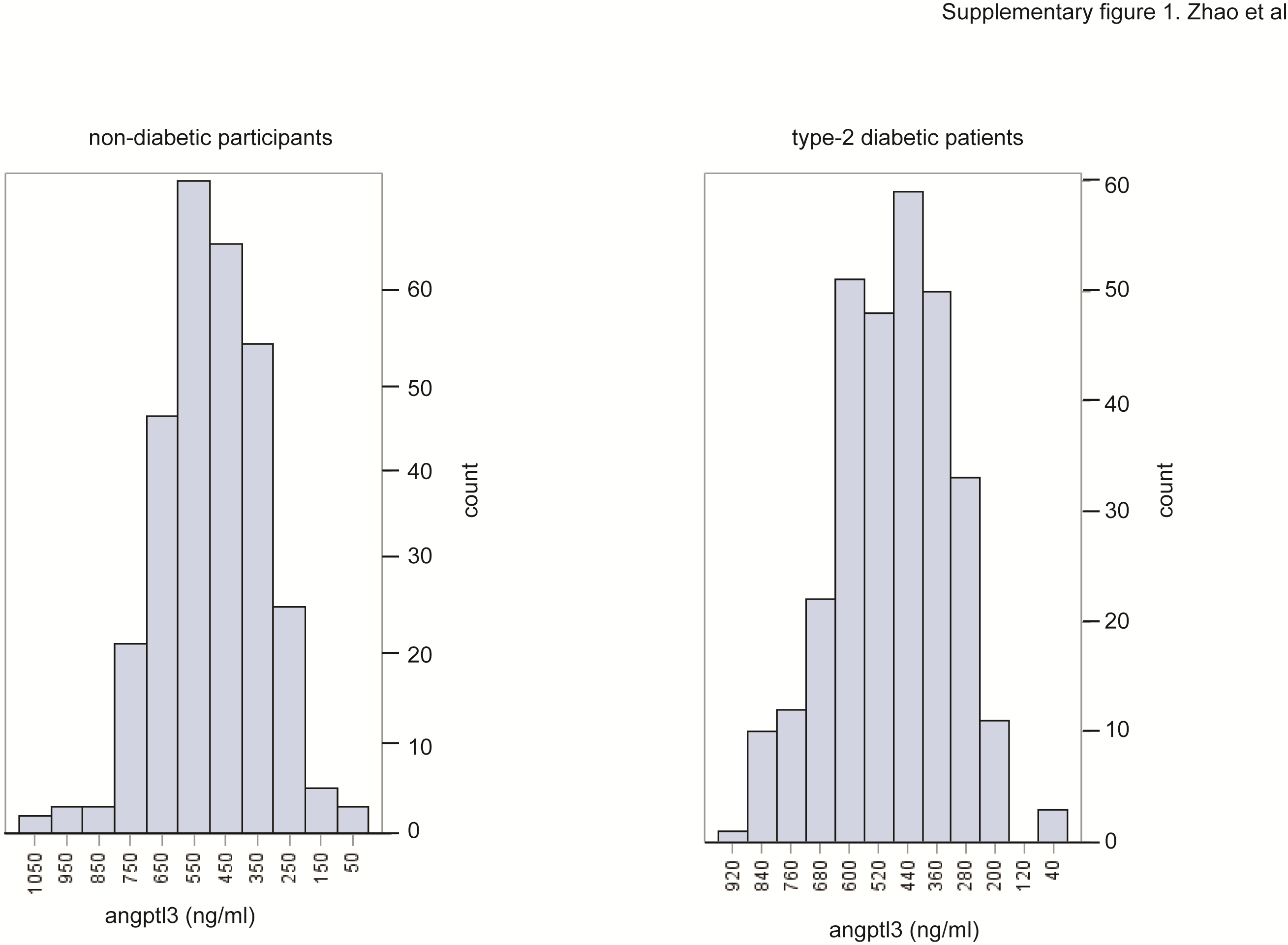


**Supplementary figure 1. Distribution and probability plot of plasma level of ANGPTL3 in the entire non-diabetic participants (A) and type-2 diabetic patients (B).**

**Supplementary table 1.** **Covariables selected by stepwise regression in non-diabetic participants and T2DM patients.**

| variables |  | Non-diabetic | | |  | T2DM | | |
| --- | --- | --- | --- | --- | --- | --- | --- | --- |
|  |  | β |  | p |  | β |  | p |
| R2 |  | 0.19 |  |  |  | 0.66 |  |  |
| Age (year) |  | 1.68 |  | 0.11 |  | 1.62 |  | 0.11 |
| Sex (0, 1) |  | 135.82 |  | <0.0001 |  | NA |  | NA |
| Mean arterial pressure (mmHg) |  | 1.25 |  | 0.06 |  | 0.04 |  | 0.03 |
| Use of medications |  |  |  |  |  |  |  |  |
| Lipid-lowering (0, 1) |  | NA |  | NA |  | -58.05 |  | 0.08 |
| vasodilators (0, 1) |  | 52.02 |  | 0.10 |  | -80.70 |  | 0.0002 |

In the stepwise regression analyses, p‑values for entering and retaining covariables in the models were set at 0.15. Covariables considered included sex, age, body mass index, mean arterial pressure, lipid-lowering drugs (statins and niacin), antihypertensive drugs vasodilators [calcium channel blockers and α blockers] and inhibitors of renin-angiotensin system [β blockers, angiotensin-converting enzyme inhibitors and angiotensin receptor blockers]. Body mass index was calculated by body weight (in kilogram) divided by the square of height (in meter); Mean arterial pressure = ((2*diastolic blood pressure) + systolic blood pressure)/3; NA indicates the covariable that did not enter the model because of its p value higher than 0.15; β indicates regression coefficient.
